# Heated air humidification versus cold air nebulization in newly tracheostomized patients

**DOI:** 10.1002/hed.24917

**Published:** 2017-10-09

**Authors:** Richard Birk, Alexander Händel, Angela Wenzel, Benedikt Kramer, Christoph Aderhold, Karl Hörmann, Boris A. Stuck, J. Ulrich Sommer

**Affiliations:** ^1^ Department of Otorhinolaryngology, Head and Neck Surgery University Hospital Marburg, Philips‐Universität Marburg Germany; ^2^ Department of Otorhinolaryngology, Head and Neck Surgery University Hospital Mannheim, Ruprecht‐Karls Universität Heidelberg Germany

**Keywords:** humidification, nurse care, tracheostomy, tracheostomy care ciliary beat frequency (CBF)

## Abstract

**Background:**

After tracheostomy, the airway lacks an essential mechanism for warming and humidifying the inspired air with the consequent functional impairment and discomfort. The purpose of this study was to compare airway hydration with cold‐air nebulization versus heated high‐flow humidification on medical interventions and tracheal ciliary beat frequency (CBF).

**Methods:**

Newly tracheostomized patients (n = 20) were treated either with cold‐air nebulization or heated humidification. The number of required tracheal suctioning procedures to clean the trachea and tracheal CBF were assessed.

**Results:**

The number of required suctions per day was significantly lower in the heated humidification group with medians 3 versus 5 times per day. Mean CBF was significantly higher in the heated humidification group (6.36 ± 1.49 Hz) compared to the cold‐air nebulization group (3.99 ± 1.39 Hz).

**Conclusion:**

The data suggest that heated humidification enhanced mucociliary transport leading to a reduced number of required suctioning procedures in the trachea, which may improve postoperative patient care.

## 1 INTRODUCTION

After tracheostomy, relevant functions of the upper airway cease to exist. One major function of the upper airway is conditioning of the inspired air to body temperature and 100% relative humidity.^1^, ^2^ Consequently, tracheostomy can lead to pathological changes of the lower airways, including damage to the ciliated tracheal mucosa, thickening of airway secretions, and the loss of mucociliary transport.^3^ As a result, patients complain about crust and mucus plug formation in the lower airway, particularly in the early postoperative period after tracheostomy. Repeated cleaning and suctioning of the lower airway/the trachea is necessary, which results in significant patient discomfort and increases the risk of lower respiratory tract infection and airway obstruction.

Ciliated epithelial cells are the basis of mucociliary transport in the upper and lower airway. Their primary task is the elimination of dust and inhaled particles by transporting debris in a layer of mucus with a fast and synchronous ciliary beat frequency (CBF), and related mucociliary clearance are influenced by many factors, including disease, medication, long‐term exposure to nicotine or alcohol, and even temperature and humidity.^4^, ^5^, ^6^, ^7^, ^8^, ^9^, ^10^

The pivotal role of a functionally intact mucosa in the lower airway supports the use of heated and humidified air delivered through tracheostomy.^3^ To ensure this conditioning of inspired air, different techniques are used. Heated humidifiers, heated and non‐heated water nebulizers, and passive humidifiers are commonly applied.^11^ A common method after tracheostomy is the use of heat‐and‐moisture exchangers (HMEs), also referred to as “artificial noses.” They are passive humidifiers that retain heat and moisture from the expired air of the patient and return a part of it after inspiration. For mechanically ventilated patients, HMEs are often used due to their low cost.^12^ Spontaneously breathing patients with long‐term tracheostomies who use these devices have significantly fewer complaints of sputum production and coughing and report a better quality of life.^13^, ^14^ The most efficient but also most expensive heated humidifiers are those that channel the airflow through a heated water bath before inspiration.^12^ With this technique, 100% relative humidity and natural body temperature of inspired air can be achieved.

Despite the paramount role of airway conditioning in patients after tracheostomy, however, comparative data regarding the objective and subjective effects of different techniques of airway humidification is rarely available, especially in regard to ciliary function. The present study provides data comparing nonheated (cold air) water nebulization and heated humidification on cilia activity and nursing care in newly tracheostomized patients.

## 2 MATERIALS AND METHODS

The study was performed at the Department of Otorhinolaryngology, Head and Neck Surgery, at the University Hospital Mannheim, Germany. The local ethics board of the Medical Faculty Mannheim, University of Heidelberg, reviewed and approved the protocol (reference number 2013‐402M‐MA). The authors obtained written informed consent from all participants. Included in the study were 20 adult patients, predominantly with head and neck malignancies, who underwent tracheostomies for any reason. Exclusion criteria were a known ciliary dysfunction or a previous tracheostomy.

### 2.1 Study protocol

Patients for the study were recruited before or within the first 24 hours after tracheostomy. After informed consent, patients were divided into 2 groups; one group received cold‐air nebulization and the other heated humidification, according to availability. Randomization was not performed. The cold‐air nebulization group received a Cirrus nebulizer set (Intersurgical, Wokingham, UK), which was connected to the compressed air supply at ambient temperature, and the flow was set to 8 L/min. The heated humidification group received an AIRVO 2 humidifier (Fisher & Paykel Healthcare, Auckland, New Zealand). An air‐flow of 30 L/min was used, and the temperature was set to 37°C.

The patients were instructed to use the humidifiers at least 8 hours per day. An exchange of the tracheal cannula was performed according to the standard operating procedure on days 2, 4, 6, 8, and 10 after surgery. After 14 days, the study documentation and ciliary experiments described below ended, but patients could use their devices during the entire clinical stay.

### 2.2 Tracheostoma care and tracheal suction

Specially trained nurses performed tracheostoma and tracheal care (suctioning, removing of crusts, etc). The suction procedure was based on current knowledge. A suction catheter was gently inserted into the tracheostoma and the trachea for a maximum of 5.9 inches (15 cm) or until resistance was detected, for a maximum of 10 seconds.^15^, ^16^ The suctioning procedure was performed according to clinical needs (airway obstruction due to crust formation or mucus retention, as indicated by the patient or detected by the nursing staff trained in tracheostomy management). Suction procedures were documented on a daily chart for up to 14 days.

### 2.3 Ciliary beat frequency

The CBF in tracheal epithelial cells was measured on days 2, 4, 6, 8, and 10 after the tracheostomies. The ciliated samples were harvested by brushing the trachea with a standard cytology brush (Gynobrush Plus; Heinz Herenz, Hamburg, Germany), which was dipped in a 0.9% saline solution before brushing.^8^ The patient was placed in a sitting position on an examination chair and the cannula was removed. The brushing was performed 2 cm below the previous location of the cannula to ensure that the mucosa in the area of cell harvesting was not damaged directly by the cannula. After the brushing, a new cannula was inserted into the patient's tracheostoma. Ex vivo analysis of ciliary function was performed in an observer‐blind manner. Directly after brushing, CBF was analyzed, as previously described.^8^ Cells were dispensed by dunking and twisting the brush in 5 mL of Roswell Park Memorial Institute medium (Roswell Park Memorial Institute 1640, cell culture tested, standard, L‐glutamine: 300 mg/L; PromoCell, Heidelberg, Germany), which was heated up to 22°C. Cells were transferred into a Petri dish and placed under an inverted phase‐contrast microscope (Leica Microsystems GmbH, Wetzlar, Germany) to visualize them under 400‐fold magnification. Five to 10 sequences of 2 seconds at a rate of 100 frames per second were recorded using a high‐speed digital camera with the Sisson‐Ammons Video Analysis software. The CBF was analyzed with the Sisson‐Ammons Video Analysis software system's region of interest method. Therefore, a rectangular area containing active beating cilia was selected to analyze the recurring bright/dark changes.^17^ As the ciliary motor function is highly temperature‐dependent,^18^ experiments were performed in a stable, temperature‐controlled environment at 22°C.

### 2.4 Statistical analysis

Statistical analysis and plotting was done using “R” as an open source statistical environment using the Mann‐Whitney *U* test.^19^ The data for the suctioning procedure did not pass the normality test, so a Mann‐Whitney Wilcoxon test was used for statistical analysis. The data for CBF was normally distributed and an unpaired *t* test was used to compare means. All data were corrected for repeated measurements by the Holm's method.^20^

## 3 RESULTS

Twenty patients were initially included, and 18 patients finished the study as intended (12 men/6 women; median age was 70 years with a range of 16). One patient (in the heated humidification group) transferred to the intensive care unit due to postoperative myocardial infarction, making it impossible to follow the study protocol; the other patient (in the cold‐air nebulization group) was discharged before termination of the study. All patients but 1 were tracheostomized due to malignancies of the head and neck area, including 17 patients with squamous cell carcinoma and 1 patient with lymphoma (CD‐20 positive high‐grade B‐cell lymphoma). One patient was tracheostomized temporarily for severe obstructive sleep apnea with intolerance to the continuous positive airway pressure machine. All patients but 1 were active smokers (at least 20 pack‐years). Chronic obstructive pulmonary disease or other pulmonary diseases were not documented in the clinical history in any patient. There were no statistically significant differences between the 2 groups for age, sex, or body mass index. During the study, there were no adverse events, such as tracheostoma occlusion, hemorrhage, fever, lower or upper respiratory infection, episodes of hypoxia, or death. One myocardial infarction, however, occurred in a patient in the heated humidification group. Further details regarding the final study cohort are shown in Table 1.

**Table 1:** Overview of included patients

	Sex	BMI	Age, years	Diagnosis
Cold‐air nebulization group				
1	M	19.6	52	T2N2bM0 laryngeal SCC
3	M	15.2	48	T2N0M0 laryngeal SCC
5	F	23.8	63	T4bN2bM0 oropharyngeal SCC
7	F	30.9	58	T3N2bM0 oropharyngeal SCC
9	M	26	88	T3N0M0 laryngeal SCC
11	M	33.6	56	Obstructive sleep apnea
13	M	19.5	59	T4aN2b laryngeal SCC
15	F	33.4	74	B‐cell lymphoma
17	M	25.7	55	T3N2bM0 hypopharyngeal SCC
Heated humidification group				
2	M	19.6	70	T3N1M0 oropharyngeal SCC
4	M	15.2	68	T3N2bM0 laryngeal SCC
6	M	23.7	74	T3N1M0 Larynx SCC
8	M	23.4	74	T3N1M0 Larynx SCC
10	M	22.8	70	T3N0M0 Laryngeal SCC
12	F	23.6	58	T3N2M0 Hypopharyngeal SCC
14	F	25	71	T4bN2bM0 Hypopharyngeal SCC
16	M	27.1	69	T4aN2cM0 oropharyngeal SCC
18	F	16.9	65	T2N2cM0 laryngeal SCC

Abbreviations: BMI, body mass index; SCC, squamous cell carcinoma.

### 3.1 Counting suction procedures

**Figure 1:**
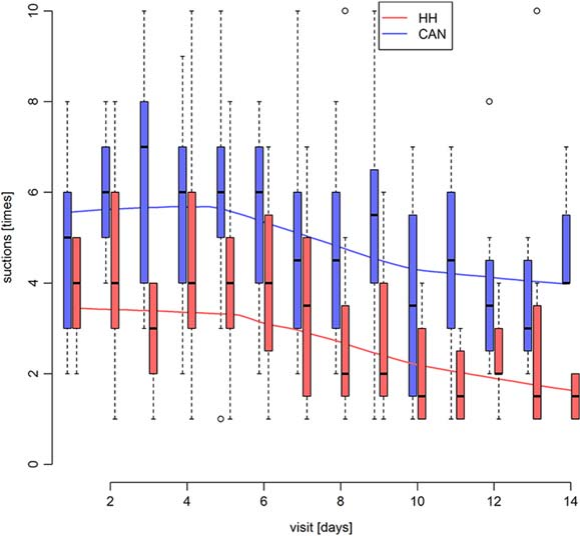
Box‐and‐whisker plot of suction procedures in the cold‐air nebulization (CAN) group and the heated humidifier (HH) group. The boxes represent the interquartile range (IQR), with the whiskers extending up to 1.5 times the IQR. The median is marked with a solid line. Outliers are marked with a circle. Visits are shown in days, and suction in necessary numbers [Color figure can be viewed at wileyonlinelibrary.com]

The number of required manual suction procedures at the trachea was higher in the cold‐air nebulization group compared with the heated humidification group during the study. The data did not pass the normality test, which is most likely due to the nature of the parameter. The number of suction procedures can be small but never <0 (realistically 1), whereas some patients would require 10 or more procedures per day causing skewness of the raw data in both groups. The overall number of required suction procedures continuously decreased from days 5 to 14 in both groups (Table 2) with statistically significant difference (*P* < .05) on days 9, 11, and 14. After correction by the Holm's method for repeated measurements, there were no statistical differences. The overall median number of tracheal suctions per day in the cold‐air nebulization group was 5 with a range of 12 from 1 to 13 (total number of suctions = 117). The median number of tracheal suctions per day in the heated humidification group was 3 with a range of 13 from 1 to 14 (total number of suctions = 116). The results report a significantly higher (*P* < .001) number of required suction procedures over 14 days in the cold‐air nebulization group compared with the heated humidification group (see Figure 1).

**Table 2:** Median numbers of necessary tracheal suction procedures per day in the cold‐air nebulization and heated humidifier groups

	Cold‐air nebulization group	Heated humidification group	
Day	Median	Median	*P* value
1	5.0	4.0	.1183
2	6.0	4.0	.1145
3	7.0	3.0	.0955
4	6.0	4.0	.2665
5	6.0	4.0	.1058
6	6.0	4.0	.1266
7	4.5	3.5	.3409
8	4.5	2.0	.0606
9	5.5	2.0	.0488
10	3.5	1.5	.1562
11	4.5	1.5	.011
12	3.5	2.0	.0862
13	3.0	1.5	.1585
14	4.0	1.5	.0002

*P* value is per day. After Holm's correction before repeated measurements there was no statistical significance.

### 3.2 Ciliary beat frequency

**Figure 2:**
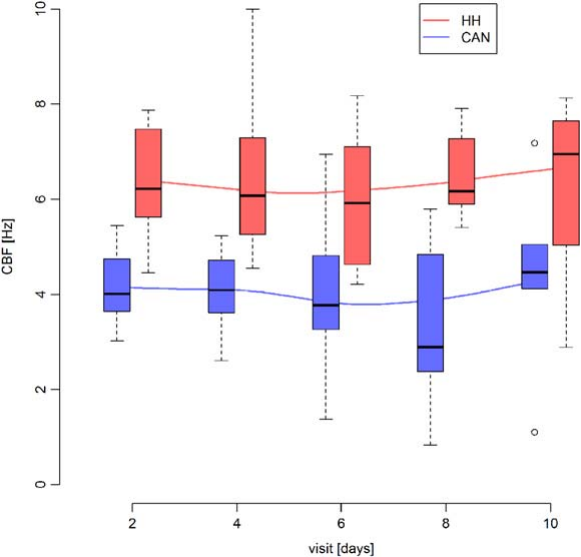
Box‐and‐whisker plot of ciliary beat frequency (CBF) changes in the cold‐air nebulization (CAN) group and the heated humidifier (HH) group. The boxes represent the interquartile range (IQR), with the whiskers extending up to 1.5 times the IQR. The median is marked with a solid line. Outliers are marked with a circle. Visits are shown in days, and CBF in Hz [Color figure can be viewed at wileyonlinelibrary.com]

The CBF was lower in patients receiving cold‐air nebulization compared to the heated humidification group at all time points. On day 2, CBF was 4.2 ± 0.8 in the cold‐air nebulization group and 6.4 ± 1.2 in the heated humidification group, and the values remained stable during the 10‐day period (for details, see Table 2). After correction by the Holm's method for repeated measurements,^20^ the differences in CBF between the 2 groups were statistically highly significant (*P* < .01) on days 2 and 8, and significant (*P* < 0.05) on days 4 and 6. Overall, the mean CBF in the cold‐air nebulization group was 3.99 ± 1.39 Hz and in the heated humidification group it was 6.36 ± 1.49 Hz (*P* < .001). Figure 2 provides graphical interpretation.

**Table 3:** Ciliary beat frequency (Hz) in the cold‐air nebulization and heated humidifier groups

	Heated humidification group		Cold‐air nebulization group		
Day	Mean	SD	Mean	SD	*P* value
2	6.4	1.2	4.2	0.8	.004
4	6.5	1.7	4.1	0.9	.02
6	6.0	1.5	4.1	1.6	.046
8	6.8	1.4	3.4	1.7	.004
10	6.3	1.8	4.4	2.0	.09

*P* value is after Holm's correction for repeated measurements.

## 4 DISCUSSION

A significant number of patients receive a tracheotomy for any reason. For example, >100 000 tracheostomies are performed annually in the United States.^21^ Across Europe, 7%‐16% of critical care admissions are managed with a tracheostomy, similar to data from the United States.^22^ Around 25% of patients with upper aerodigestive tract malignancies, such as head and neck cancers, receive a tracheostomy.^23^ The resulting stoma allows patients to breathe, bypassing the upper airway. Consequently, humidification of and heating the inspired air is compromised. In clinical routine, heated humidifiers, jet nebulizers, and HMEs are used for compensation. The HMEs show wide variation in water exchange performance (range 0.5‐3.6 mg/0.5 L air)^24^ and are the cheapest systems to use.^12^ Jet nebulizers can be efficient in delivering aerosolized solutions to the lungs and trachea in mechanically ventilated patients, but in spontaneously breathing patients, a heated humidifier and HME have better humidification and thermic capacities.^25^, ^26^ Nebulizers are also reported to be potentially harmful. In rabbits, long‐term exposure to ultrasonic nebulized saline (72 hours) led to pathological pulmonary changes comparable to severe bronchopneumonia.^27^ Additionally, in patients with chronic bronchitis, a significant decrease in 1‐second forced expiratory volume and vital capacity has been observed after saline inhalation via ultrasonic nebulizer.^28^ The advantage of heated humidifiers over HMEs has been shown previously. Heated humidifiers decrease adverse clinical events in children over a 10‐month period.^29^ Moreover, in intubated patients without a tracheostomy, heated humidification systems tested superior to HMEs. Fewer tracheostomies were needed; there was less hypothermia, and fewer thick, tenacious bronchial secretions were noted.^30^

In this study, the number of suction procedures and manual interventions required to clean the upper airway could be reduced in the heated humidification group by 40% from median value, 5 suction procedures per day to 3 per day. One explanation for this could be that there was higher CBF measured in this group. The mean overall CBF increased from 3.99 Hz ±1.39 in the cold‐air nebulization group to 6.36 Hz ±1.49, which corresponds to an increase of 37%. The CBF difference was also statistically significant on measurement days 2, 4, 6, and 8, but showed weak correlation with the number of suctioning procedures, but the data on correlation were not presented.

The impact of different techniques of airway humidification on tracheal epithelial CBF in tracheostomized patients has not been evaluated to date. In the present study, 20 newly tracheostomized patients, mostly due to head and neck cancer surgeries, were treated with cold‐air nebulization or heated humidification for 14 days, and CBF as well as tracheostomal suctions were assessed. The CBF measurements showed a significantly higher ciliary activity in patients supplied with the heated humidification systems in comparison to the cold‐air nebulizing systems. The normal CBF of tracheal epithelial cells collected during bronchoscopy is 11.3 beats/second when measured at a temperature of 23‐25°C. A CBF of 5.7 ± 2.5 Hz was shown in a comparable study with nasal epithelial cells using the same methodology for CBF assessment.^31^ The presented data, therefore, could be interpreted as an impaired ciliary function in both groups, which could be explained by epithelial irritation caused by the tracheostomy. An alternative explanation could be that harvesting the ciliated cells was performed with a cytobrush, in contrast to using a biopsy during bronchoscopy. With regard to the significantly lower CBF in the cold‐air nebulization group compared to the heated humidification group, as well as to the nasal CBF in healthy subjects, an impaired ciliary function in the cold‐air nebulization group could be demonstrated. Increased viscoelasticity in the trachea mucus layer in cold‐air nebulization could explain the impaired ciliary function, similar to increased viscoelasticity in cystic fibrosis.^32^ Optimal mucociliary clearance depends on the ability of the airway epithelium to hydrate secreted mucins so that mucus concentrations are optimal for cilial‐dependent mucus transport.^33^, ^34^ Additional CBF and the associated mucociliary clearance are temperature‐dependent, the higher CBF in the heated humidification group may be attributed to the more stable physiologic airway temperature with the heated system, even if the CBF measuring was at the same temperature in cold‐air nebulization group and in the heated humidification group.^4^, ^5^, ^35^

The key strength of the study is its prospective nature and use of a clinically relevant outcome ‐ the analyses of the number of required suction procedures. The data in this study support the theory of a protective role of heated humidification on mucociliary function in the airways. The mechanisms for improved outcome could be a reduction in mucus secretion or improved mucociliary clearance resulting in less need for suctioning in newly tracheostomized patients. Another strength of this study is a comparison of standard care procedure (cold‐air nebulization) with an alternative care provided by continuous active humidification of inspired air. The most important limitation of the study is that it is a nonrandomized study and that the CBF measurement was performed at 22°C and not with a heated microscope stage. In addition, blinding with regard to the type of humidification used was not possible considering the obvious technical difference between the humidification systems. Another limitation is that the patient comfort and preference were not assessed. There is also a lack of baseline measurements in the heated humidification group to enable analyses of the effects of heated humidification in every patient individually, particularly CBF.

## 5 CONCLUSION

Heated humidification demonstrated a potential advantage over cold‐air nebulization in newly tracheostomized patients with regard to physiologic and clinically relevant parameters. The number of required suction procedures was reduced, and the CBF was higher in the heated humidification group. Results indicate a potential advantage of hydration with heated and humidified air over the conventional nebulization of cold saline solution after a tracheotomy. A larger randomized trial might be required for studying the clinical outcomes of heated humidification and comparing these with the use of HME in tracheostomized patients.

## AUTHOR CONTRIBUTIONS

*Clinical assessment and experiments:* Birk, Händel

*Writing of manuscript:* Aderhold, Birk, Kramer, Sommer, Stuck, Wenzel

*Scientific support:* Hoermann

*Study initiation and study design:* Birk, Sommer, Stuck

*Scientific and functional support:* Sommer
